# Paroxysmal Autonomic Instability with Dystonia after Severe Traumatic Brain Injury

**DOI:** 10.5334/tohm.81

**Published:** 2020-06-29

**Authors:** Thiago Cardoso Vale, Leandro Echenique, Orlando Graziani Povoas Barsottini, José Luiz Pedroso

**Affiliations:** 1 Movement Disorders Unit, Departament of Internal Medicine, Universidade Federal de Juiz de Fora, Juiz de Fora, BR; 2 Division of General Neurology and Ataxia Unit, Department of Neurology, Universidade Federal de São Paulo, Sao Paulo, BR

**Keywords:** traumatic brain injuries, dystonia, movement disorders

## Abstract

**Background:**

Paroxysmal autonomic instability with dystonia (PAID) syndrome, a subset of dysautonomia, is characterized by paroxysms of marked agitation, diaphoresis, hyperthermia, hypertension, tachycardia and tachypnea accompanied by hypertonia and extensor posturing.

**Case Report:**

We report a 52-year-old man who was severely brain injured and developed spastic tetraparesis with cognitive impairment. During his Intensive care unit stay and rehabilitation period, he presented with paroxysmal episodes of dystonic posturing accompanied by dysautonomia.

**Discussion:**

Our case raises awareness of PAID, a life-threatening condition which can mimic many others and poses significant challenges in the acute management and rehabilitation of patients.

**Highlights:**

- PAID is characterized by paroxysms of marked agitation, diaphoresis, hyperthermia, hypertension, tachycardia and tachypnea accompanied by hypertonia and extensor posturing.
- It usually presents in patients with severe brain injury primarily due to trauma or hypoxia resulting in diffuse axonal or brainstem injury.
- PAID is also associated with tuberculous meningitis, interpeduncular tuberculoma, pneumococcal meningoencephalitis, intracerebral hemorrhage and paraneoplastic limbic encephalopathy.
- Differential diagnosis of PAID include neuroleptic malignant syndrome, malignant hyperthermia, sepsis, thyroid storm, pheochromocytoma, autonomic epileptic seizures, sepsis and impending cerebral herniation.

## Introduction

Paroxysmal autonomic instability with dystonia (PAID) syndrome is characterized by episodes of agitation, dysautonomia (diaphoresis, hyperthermia, hypertension, tachycardia and tachypnea) combined with extensor posturing. PAID can easily mimic other life-threating conditions in the intensive care unit (ICU) setting, such as sepsis, impending herniation and epileptic seizures [1,2]. The main causes of PAID are severe brain injury due to trauma or hypoxia [1,2]. We report the first videotaped case of PAID syndrome secondary to a severe brain injury and then we discuss the clinical presentation, pathophysiology, and management of this rare and under-recognized clinical entity.

**Figure 1:**
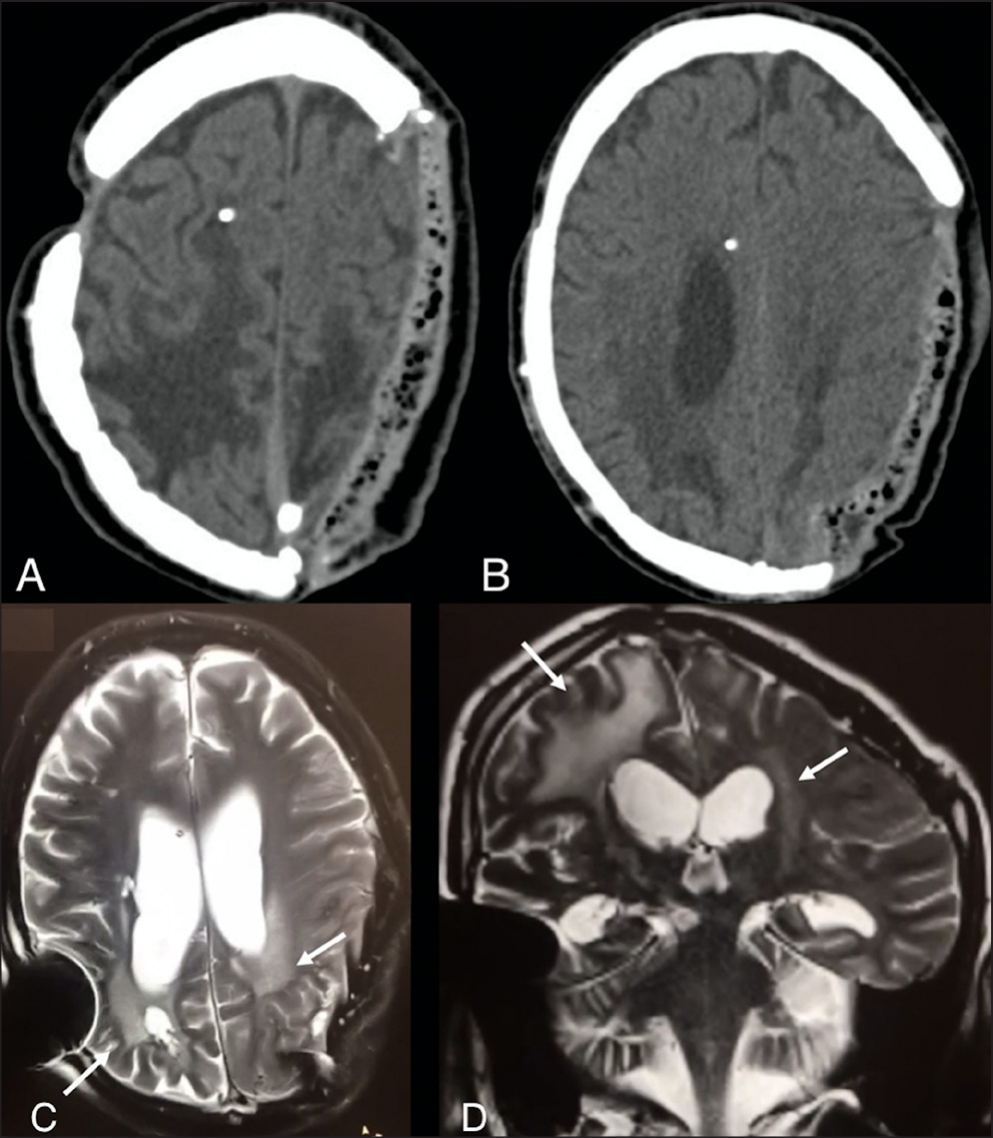
**A–B** Cranial computed tomography scan showing an extensive left parietal craniotomy, trepanation in the right coronal suture with an external ventricular drain placed in the frontal horn of the right lateral ventricle. Multiple skullcap fractures with misalignment mainly in the right parietal region. High convexity biparietal hypodense lesions with extension to the frontal lobes and adjacent deep white-matter with compensatory lateral ventricles enlargement. **C–D:** (C) Axial T2-weighted and (D) coronal T2-weighted brain magnetic resonance imaging showing diffuse brain lesions involving the white matter and cortex, particularly in the posterior lobes, related to the previous cranial trauma (white arrows).

A 52-year-old man was severely brain injured in an accident that required neurosurgical intervention and ventricular drainage (Figure 1A–D) and developed spastic tetraparesis with cognitive impairment. During his ICU stay and throughout a year of his rehabilitation period, he developed paroxysmal episodes of dystonic posturing accompanied by tachycardia, tachypnea, hypertension and diaphoresis (Video 1). Most episodes lasted for a few minutes, but eventually some longer-lasting ones occurred. He gradually recovered in use of gabapentin, clonazepam and clonidine. There was no continuous use of atypical neuroleptics, bromopride, or metoclopramide that could induce dystonic posture. Video electroencephalogram in several occasions ruled out seizures or epileptic discharges during dystonic posture. After a thorough investigation, he was diagnosed with PAID.

**Video 1:** **Paroxysmal Autonomic Instability with Dystonia.** Video shows four bouts of dystonic posturing in a semi-opisthotonic pattern combined with dyspnea, tachycardia and hypertension (the last two are not shown on video).

PAID syndrome includes the following signs and symptoms: raised temperature (≥38.5°C), tachycardia (≥130 beats per minute), tachypnea (≥140 breaths per minute), agitation (Rancho Los Amigos Scale level ≤IV), diaphoresis and dystonia (rigidity or decerebrate posturing) [1,2]. The cyclic episodes usually occurs within weeks of the brain injury and can last minutes to hours, with at least one cycle per day. It can persist for weeks or months, particularly if the cause was hypoxic-ischemic.

PAID syndrome mainly manifests in the ICU but may persist for months during the patient’s rehabilitation phase [1,2]. Apart from trauma and anoxic injury, PAID is associated with tuberculous meningitis [3], interpeduncular tuberculoma [4], pneumococcal meningoencephalitis [5], intracerebral hemorrhage [6] and paraneoplastic limbic encephalopathy [7]. Differential diagnosis of PAID include neuroleptic malignant syndrome, malignant hyperthermia, sepsis, thyroid storm, pheochromocytoma, autonomic epileptic seizures, sepsis and impending cerebral herniation. Another important differential diagnosis is dystonic storms, which is a hyperkinetic movement disorder emergency, characterized by fever, tachycardia, tachypnea, hypertension, sweating and autonomic instability, with the potential to progress to dysarthria, dysphagia and respiratory failure. It usually occurs in patients with known dystonia, either genetic or acquired, which was not the case of our patient. In addition, dystonic storms tend to last longer than the paroxysmal nature of PAID.

PAID pathophysiology is still uncertain, but best explained by dysfunction of autonomic centres in the diencephalon or their connections to cortical, subcortical and brainstem loci that mediate autonomic function [1,2]. Boeve et al. [8] speculated that PAID syndrome might be the result of the activation or disinhibition of central sympathetic excitatory regions in the brain such as the paraventricular hypothalamic nucleus, lateral periaqueductal grey substance, lateral parabrachial nucleus or rostral ventricular medulla.

Treatment of PAID is aimed at managing the dysautonomia and the hypertonia. Medications such as morphine, clonazepam, non-selective beta-blockers, baclofen, bromocriptine, dantrolene, clonidine and gabapentin have all been tried with anecdotal successful cases [9]. Goddeau et al. [10] achieved complete resolution of PAID symptoms after a 72-hour infusion of dexmedetomidine followed by clonidine. In several traumatic brain injury-induced PAID syndrome, symptoms resolved as the intracranial pressure normalized. Alcohol neurolysis and botulinum toxin type A injection were also proposed as treatment options for intractable PAID [7].

In our patient, there were 2–3 paroxysms of symptoms that lasted for approximately 30 minutes and subsided spontaneously. In most cases, episodes lasted for only a few minutes. The patient was free of any additional symptoms or signs in between the paroxysms, returning to his usual baseline. After a combined treatment of clonazepam, gabapentin and clonidine, episodes were fewer and shorter until resuming after a year of the trauma.

In conclusion, PAID poses significant challenges in the acute management and rehabilitation of the patients. We think this is an underdiagnosed condition, even by movement disorders specialists, although no data exist regarding how often this diagnosis is missed. Early recognition of this syndrome would permit adequate treatment and alleviate physicians concern of overseen a life-threatening condition.
